# Identifying regulational alterations in gene regulatory networks by state space representation of vector autoregressive models and variational annealing

**DOI:** 10.1186/1471-2164-13-S1-S6

**Published:** 2012-01-17

**Authors:** Kaname Kojima, Seiya Imoto, Rui Yamaguchi, André Fujita, Mai Yamauchi, Noriko Gotoh, Satoru Miyano

**Affiliations:** 1 Human Genome Center, Institute of Medical Science, University of Tokyo, 4-6-1 Shirokanedai, Minato-ku, Tokyo 108-8639, Japan; 2 Institute of Mathematics and Statistics, University of São Paulo, Rua do Matão, 1010, São Paulo 05508-090, Brazil

## Abstract

**Background:**

In the analysis of effects by cell treatment such as drug dosing, identifying changes on gene network structures between normal and treated cells is a key task. A possible way for identifying the changes is to compare structures of networks estimated from data on normal and treated cells separately. However, this approach usually fails to estimate accurate gene networks due to the limited length of time series data and measurement noise. Thus, approaches that identify changes on regulations by using time series data on both conditions in an efficient manner are demanded.

**Methods:**

We propose a new statistical approach that is based on the state space representation of the vector autoregressive model and estimates gene networks on two different conditions in order to identify changes on regulations between the conditions. In the mathematical model of our approach, hidden binary variables are newly introduced to indicate the presence of regulations on each condition. The use of the hidden binary variables enables an efficient data usage; data on both conditions are used for commonly existing regulations, while for condition specific regulations corresponding data are only applied. Also, the similarity of networks on two conditions is automatically considered from the design of the potential function for the hidden binary variables. For the estimation of the hidden binary variables, we derive a new variational annealing method that searches the configuration of the binary variables maximizing the marginal likelihood.

**Results:**

For the performance evaluation, we use time series data from two topologically similar synthetic networks, and confirm that our proposed approach estimates commonly existing regulations as well as changes on regulations with higher coverage and precision than other existing approaches in almost all the experimental settings. For a real data application, our proposed approach is applied to time series data from normal Human lung cells and Human lung cells treated by stimulating EGF-receptors and dosing an anticancer drug termed Gefitinib. In the treated lung cells, a cancer cell condition is simulated by the stimulation of EGF-receptors, but the effect would be counteracted due to the selective inhibition of EGF-receptors by Gefitinib. However, gene expression profiles are actually different between the conditions, and the genes related to the identified changes are considered as possible off-targets of Gefitinib.

**Conclusions:**

From the synthetically generated time series data, our proposed approach can identify changes on regulations more accurately than existing methods. By applying the proposed approach to the time series data on normal and treated Human lung cells, candidates of off-target genes of Gefitinib are found. According to the published clinical information, one of the genes can be related to a factor of interstitial pneumonia, which is known as a side effect of Gefitinib.

## Background

Gene network estimation from time series gene expression data is a key task for elucidating cellular systems. Thus far, wide variety of approaches have been proposed based on the vector autoregressive (VAR) model [1,3], the state space model [4-6], and the dynamic Bayesian network [7,8]. Recently, time series gene expression data on multiple conditions aiming at analyzing effects of cell treatment such as drug dosing and heat shock are available. We here assume that some gene regulations are disrupted but many of the gene regulations do not change due to some treatment of interest, and try to find a small number of changes on regulations as keys for elucidating effects by the treatment.

A possible way for finding changes on regulations is to estimate networks from two data sets separately and then compare their structures. However, due to the limited length of time series data (usually less than 10 time points) and unignorable measurement noise, networks are estimated with high error rates and the estimation errors cause the serious failure on identifying changes on regulations. Thus, approaches using two time series data in an efficient manner are strongly demanded. Also, widely used statistical methods such as the VAR model and dynamic Bayesian network assume equally spaced time points in time series data. However, observed time points on usually available time series data are not equally spaced [5,6,9], and approaches that can handle unequally spaced time series data in a theoretically correct way should be considered.

We propose a new statistical model that estimates gene networks on two different conditions in order to identify changes on regulations between the conditions. As the basis of the proposed model, we employ the state space representation for VAR model (VAR-SSM), in which observation noise is considered between the measured or observed gene expressions and the true gene expressions in observation model and gene regulations between true gene expressions are considered in the system model [10]. The VAR-SSM can handle unequally spaced time series data by ignoring observation model on the non-observed time points. For considering the changes on regulations, we introduce hidden variables to the VAR-SSM in order to indicate the presence of regulations in each condition. If hidden binary variables on two conditions indicating the presence of a regulation are both estimated as one, the regulation is considered as a commonly existing regulation. On the other hand, if only one of the hidden binary variables for the regulation is estimated as one, the regulation is considered as a condition specific regulation. We also introduce a potential function between the hidden binary variables that is designed to take high probability if the hidden binary variables on two conditions take the same value. From the design of the potential function, the similarity of networks on two conditions is automatically considered. Since the time series data on both conditions are used for estimating commonly existing regulation due to the use of the hidden binary variables, an efficient data assignment is achieved. In addition, from the more accurate estimation of commonly existing regulations by the efficient data assignment, accurate identification of changes on regulations is induced.

The hidden binary variables are estimated by searching the configuration of binary variables that maximizes the marginal likelihood of the model. However, searching the optimal configuration is computationally intractable. Thus, as an alternative approach, we derive a new variational annealing method based on [11] in order to estimate the hidden binary variables. We also give a proof for the effectiveness of the variational annealing compared to other candidate alternatives, the variational annealing and the EM algorithm, in order to show the validity of using the variational annealing.

For the performance evaluation, we generate two regulatory networks in such a way that most of the regulations commonly exist and some exist only on one of the networks. We then apply our proposed approach and existing var model based and dynamic Bayesian network based approaches to two equally spaced time series data drawn separately from the generated networks. From the comparisons of true positive rates and false positive rates of these approaches, we confirm the effectiveness of our approach. We also generate unequally spaced time series data from these networks, and show that our approach works correctly on unequally spaced time series data while the performance of the existing approaches assuming equally spaced time points is drastically worsened.

Our proposed approach is used to analyze changes on regulations in gene networks between normal Human lung cells and Human lung cells treated by stimulating EGF-receptors and dosing an anticancer drug termed Gefitinib. A lung cancer condition is simulated by the stimulation of EGF-receptors in the treated cells. Since Gefitinib is known as a selective inhibitor of EGF-receptors, the stimulation of EGF-receptors would be counteracted by Gefitinib, and hence the treated cells are expected to be the same condition as normal cells. However, gene expression profiles from normal and treated cells are actually different, and off-targets of Gefitinib causing unexpected positive or negative effects are implied. We focus on genes with changes on regulations between the networks estimated by our approach and find possible off-target genes of Gefitinib. According to the published clinical information, one of the possible off-target genes is suggested as one of factors of interstitial pneumonia, which is known as a side effect of Gefitinib.

## Methods

### Vector autoregressive model and its state space representation

#### Vector autoregressive model

Given gene expression profile vectors of *p *genes during *T *time points {***y***_1_, ..., ***y***_*T*_}, the first order vector autoregressive (VAR(1)) model at time point *t *is given by

$$y_{t}=Ay_{t-1}+\varepsilon_{1},$$

where *A *is a *p *× *p *autoregressive coefficient matrix, and *ε*_*t *_is observation noise at time *t *and follows $N\left(0,\text{diag}\left[\sigma_{1}^{2},...,\sigma_{p}^{2}\right]\right)$, a normal distribution with mean 0 and variance $\text{diag}\left[\sigma_{1}^{2},...,\sigma_{p}^{2}\right]$. The (*i*, *j*)th element of *A*, *A*_*ij*_, indicates a temporal regulation from the *j*th gene to the *i*th gene, and if *A*_*ij *_≠ 0, regulation from the *j*th gene to the *i*th gene is considered. By examining whether *A*_*ij *_is zero or not for all *i *and *j*, a gene network is constructed. Since equally space time points are assumed in the VAR model, it has difficulty on handling unequally spaced time series data.

#### State space representation of VAR model (VAR-SSM)

Let T be the set of equally spaced entire *T *time points and $T_{obs}$ the set of time points where gene expressions are observed. Note that $T_{obs}\subseteq T$ holds. VAR-SSM is comprised of two models: system model and observation model. Let ***x***_*t *_be hidden variable vector representing true gene expression at time *t*. The system model is given as the VAR model of ***x***_*t*_:

$$x_{t}=Ax_{t-1}+\eta_{t},\;t\in T,$$

where ***η***_*t *_is the system noise normally distributed with mean 0 and variance *H *= diag[*h*_1_*,...,h*_*p*_]. The observation model represents measurement error of observed gene expression ***y***_*t *_and true gene expression ***x***_*t *_at observed time point $t\in T_{obs}$:

$$y_{t}=x_{t}+\rho_{t},\;t\in T_{obs},$$

where ***ρ***_*t *_is the observation noise normally distributed with mean 0 and variance *R *= diag[*r*_1_*,..., r*_*p*_]. Unequally spaced time series data are handled by ignoring observation model at non-observed time points.

### Joint model of VAR-SSM for two time series data

Let ${\left\{y_{t}^{\left(c\right)}\right\}}_{t\in T_{obs}^{\left(c\right)}}$ be time series gene expression data on cell condition *c*, where $T_{obs}^{\left(c\right)}$ is the set of observed time points on cell condition *c*. We also let $T^{\left(c\right)}$ be the set of time points from 1 to *T*^(*c*)^, where $T^{\left(c\right)}=\max\left\{t\in \overset{\left(c\right)}{\underset{obs}{T}}\right\}$. Given time series data on two types of cell conditions *c *= 1 and 2, we propose a new VAR-SSM model to estimate gene networks in the two conditions as well as identify changes on regulations between them. The model is comprised of the following two equations:

$$\begin{array}{c} x_{t}^{\left(c\right)}=A\circ E^{\left(c\right)}x_{t-1}^{\left(c\right)}+\eta_{t}^{\left(c\right)},\;t\in T^{\left(c\right)},\\ y_{t}^{\left(c\right)}=x_{t}^{\left(c\right)}+\rho_{t}^{\left(c\right)},\;t\in T_{obs}^{\left(c\right)}, \end{array}$$

where ∘ denotes the Hadamard product, *E*^(*c*) ^is a *p *× *p *binary matrix, and $\eta_{t}^{\left(c\right)}$ and $\rho_{t}^{\left(c\right)}$ are respectively system and observation noises from N(0,H) and N(0,R). In this model, the (*i*, *j*)th element $E_{ij}^{\left(c\right)}$ takes one if regulation from gene *j *to gene *i *exists on condition *c *and zero otherwise, i.e., the presence of regulations is controlled by *E*^(*c*) ^and the AR coefficient matrix *A *is commonly used in conditions 1 and 2. Changes on regulations are identified when regulations exist only in a condition.

The complete likelihood of our model, *P*(*Y*, *X*, Θ*, E*), where *Y*, *X*, Θ, and *E *are respectively the sets of $y_{t}^{\left(c\right)},x_{t}^{\left(c\right)}$, parameters, and *E*^(*c*)^, is given by the following equation:

P(Y,X,Θ,E)= ∏c=12∏t∈T(c)|H|-1∕22πp exp-12(xt(c)-A∘E(c)xt-1(c))′H-1(xt(c)-A∘E(c)xt-1(c))(1)×∏t∈Tobs(c)|R|-1∕22πp exp-12(yt(c)-xt(c))′R-1(yt(c)-xt(c))P(Θ,E),(2)(3) 

where the prior distribution *P*(*Θ*, *E*) is assumed to be factorized as

$$P\left(\Theta ,E\right)=\underset{i}{\prod }P\left(h_{i}\right)P\left(r_{i}\right)\underset{j}{\prod }P\left(A_{ij}\right)P\left(E_{ij}^{\left(1\right)},E_{ij}^{\left(2\right)},z_{ij}\right).$$

Here, *z*_*ij *_is a parameter for a potential function of $E_{ij}^{\left(1\right)}$ and $E_{ij}^{\left(2\right)}$ defined later. The prior distributions of *A*_*ij *_is given by

$$P\left(A_{ij}|h_{i},E_{ij}^{\left(1\right)},E_{ij}^{\left(2\right)}\right)=N{\left(A_{ij};0,h_{i}\cdot \alpha_{1}\right)}^{F_{ij}}N{\left(A_{ij};0,h_{i}\cdot \alpha_{0}\right)}^{1-F_{ij}},$$

where *α*_0 _and *α*_1 _are parameters controlling the shrinkage of coefficients *A *and *F*_*ij *_is a binary variable that takes 1 if $E_{ij}^{\left(1\right)}$ or $E_{ij}^{\left(2\right)}$ takes 1 and 0 otherwise, i.e., *F*_*ij *_is given by $1-\prod_{c=1}^{2}\left(1-E_{ij}^{c}\right)$. *α*_1 _is set to a large value, while *α*_0 _is set to smaller than *α*_1_. From the design of the prior for *A*_*ij*_, if $E_{ij}^{\left(1\right)}$ or $E_{ij}^{\left(2\right)}$ takes one, i.e., there exists regulation from gene *j *to *i *in condition 1 or 2, weaker prior $N\left(A_{ij};0,h_{i}\cdot \alpha_{1}\right)$ is selected and the shrinkage of the coefficients are avoided. Otherwise stronger prior $N\left(A_{ij};0,h_{i}\cdot \alpha_{0}\right)$ is selected and the sparsity of the network structures is promoted. The prior distributions of *h*_*i *_, and *r*_*i *_are given by

$$\begin{array}{c} P\left(h_{i}\right)=IG\left(h_{i};u_{0},k_{0}\right),\\ P\left(r_{i}\right)=IG\left(r_{i};v_{0},l_{0}\right), \end{array}$$

where IG represents the density function of inverse gamma distribution, *u*_0 _and *v*_0 _are the shape parameters, and *k*_0 _and *l*_0 _are the inverse scaling parameters. Under the assumption that a small number of regulations change between two conditions, we design the prior distribution for $E_{ij}^{\left(1\right)}$ and $E_{ij}^{\left(2\right)},P\left(E_{ij}^{\left(1\right)},E_{ij}^{\left(2\right)},z_{ij}\right)$ by using the following potential function between $E_{ij}^{\left(c\right)}$ for *c *= 1 or 2:

$$\frac{1}{2}\phi \left(E_{ij}^{\left(1\right)},E_{ij}^{\left(2\right)};z_{ij}\right)=\left\{\begin{array}{ccc} z_{ij} & \text{if} & E_{ij}^{\left(1\right)}\neq E_{ij}^{\left(2\right)}\\ 1-z_{ij} & & \text{otherwise} \end{array}\right).$$

In this setting, if *z*_*ij *_is small, $E_{ij}^{\left(1\right)}$ and $E_{ij}^{\left(2\right)}$ tend to take the same value and thus most of the regulations exist in both two conditions. We also introduce a prior distribution for *z*_*ij *_by beta distribution with parameters *ζ*_*i*0 _and *ζ*_*i*1_:

$$B\left(z_{ij};\zeta_{i0},\zeta_{i1}\right)=\frac{1}{B\left(\zeta_{i0},\zeta_{i1}\right)}z_{ij}^{\zeta_{i0}-1}{\left(1-z_{ij}\right)}^{\zeta_{i1}-1},$$

where *B*(·) is the beta function. Thus, the prior distribution of $E_{ij}^{\left(c\right)}$ is given by

$$P\left(E_{ij}^{\left(1\right)},E_{ij}^{\left(2\right)},z_{ij}\right)=\phi \left(E_{ij}^{\left(1\right)},E_{ij}^{\left(2\right)}\right)B\left(z_{ij};\zeta_{i0},\zeta_{i1}\right).$$

**Figure 1:**
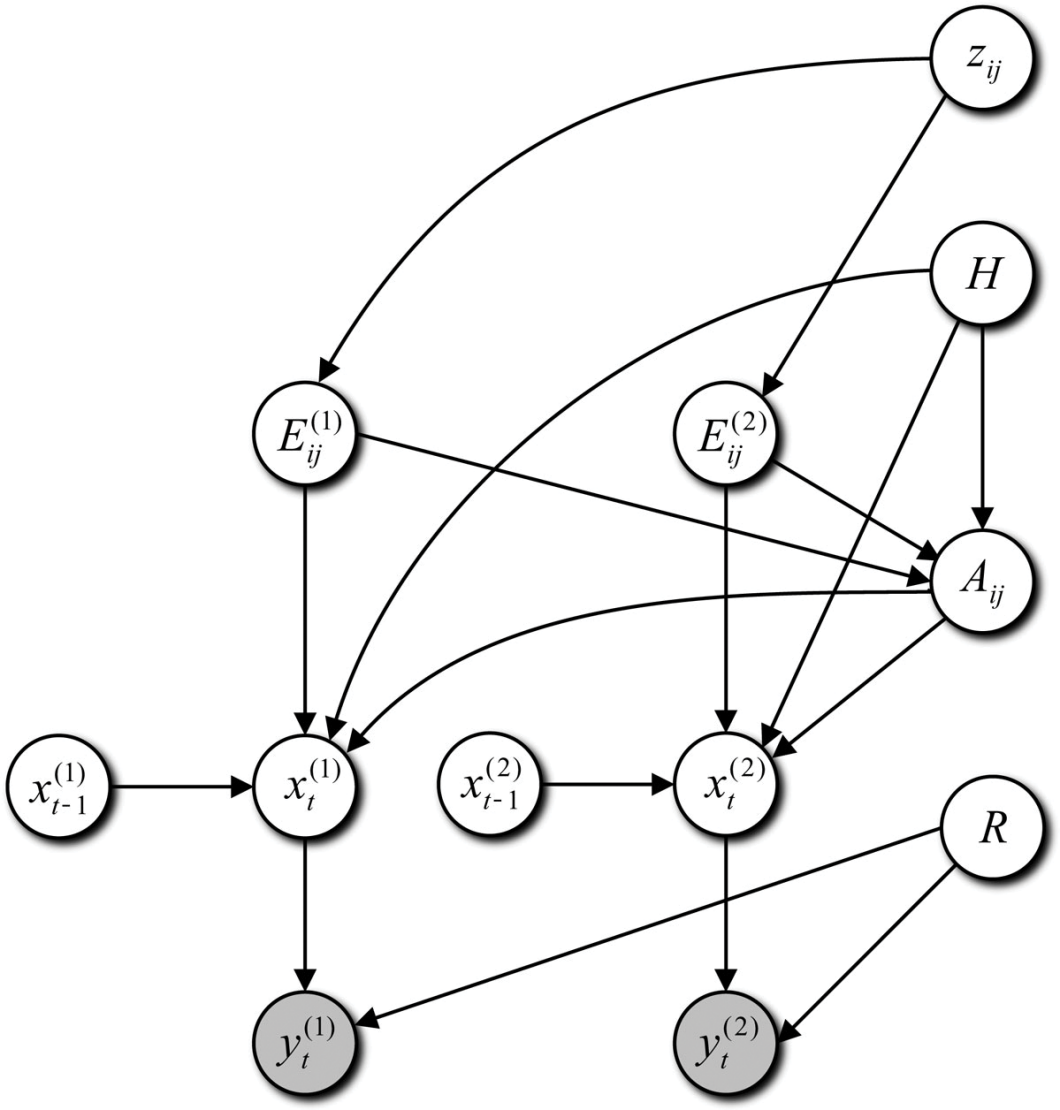
**A graphical representation of the proposed model.** Hyperparameters are omitted from this representation. The nodes in gray denote observed data.

Figure 1 shows a graphical representation of the proposed model, where dependency of the parameters and variables are indicated. The hyperparameters are omitted and the observed data ***y***_*t *_is represented by gray nodes. In the observed data $y_{t}^{\left(1\right)}$ and $y_{t}^{\left(2\right)}$ are propagated mainly via hidden variables $E_{ij}^{\left(1\right)}$ and $E_{ij}^{\left(2\right)}$. Due to the data propagation, more accurate estimation is expected in the proposed model than the approaches considering data on two conditions independently.

For the parameter estimation, we search the configuration of *E *maximizing the following marginal likelihood:

(1) $$Ê=\arg\underset{E}{\max}\int dX\int d\Theta P\left(Y,X,\Theta ,E\right).$$

Finding the optimal configuration of *E *is computationally intractable, and heuristics approaches such as the EM algorithm and the variational method are used in practice. Here, we use the variational annealing, an extension of the deterministic annealing for discrete variables [11]. In the next section, we give a small explanation of the variational annealing and show its effectiveness compared to the EM algorithm and the variational method.

### Parameter estimation by variational annealing

In the deterministic annealing, optimization problem is solved while gradually changing temperature in a some schedule, and maximum likelihood estimator is obtained like the EM algorithm [12-14]. Yoshida and West proposed to use the deterministic annealing to find the configuration of the binary variables that maximizes the likelihood of factor models with sparseness priors [11]. We derive a new variational annealing method by extending Yoshida and West's approach to find the configuration of the binary variables on marginal likelihood function, which can be applied for searching *E *that maximizes Equation (1).

Let *E*, *X*, and Θ be *p *dimensional binary variables, unobserved variables, and parameters, respectively, and consider to search *E *maximizing the following marginal likelihood:

(2) maxE∈{0,1}p log∫ dX∫ dΘP(X,Θ,E).

The maximum of the marginal likelihood on *E *is bounded by the following formula:

(3) maxE∈{0,1}p log∫ dX∫ dΘP(X,Θ,E)≥τlog∫ (0,1)pdE∫ dX∫ dΘP(X,Θ,E)1∕τ,

where *τ *is called temperature and the equality holds for *τ *→ +0. Hereafter, the integral range of *E *is omitted if no confusion occurs. Let *Q*(*E*) be a normalized non-negative function, i.e., *Q*(*E*) ≥ 0 and ∫ *Q*(*E*)*dE *= 1.

From the Gibbs inequality, the right side of Equation (3) is also bounded:

$$\tau \log\int dE{\left[\int dX\int d\Theta P\left(X,\Theta ,E\right)\right]}^{1∕\tau }\geq \int dEQ\left(E\right)\log\frac{\int dX\int d\Theta P\left(x,\Theta ,E\right)}{Q{\left(E\right)}^{\tau }}.$$

Here, *Q*(*E*) is considered as an approximation function of $\frac{P{\left(E\right)}^{1∕\tau }}{\int P{\left(E^{\prime }\right)}^{1∕\tau }dE^{\prime }}$. Under the assumption that *P*(*X*,Θ,*E*) ∝ *Q*(*X*)*Q*(Θ), where *Q*(*X*) and *Q*(Θ) are normalized non-negative functions, we have the following inequality

(4) $$\int dEQ\left(E\right)\log\frac{\int dX\int d\Theta P\left(X,\Theta ,E\right)}{Q{\left(E\right)}^{\tau }}\geq \int dE\int dX\int d\Theta Q\left(X\right)Q\left(\Theta \right)Q\left(E\right)\log\frac{P\left(X,\Theta ,E\right)}{Q\left(X\right)Q\left(\Theta \right)Q{\left(E\right)}^{\tau }}.$$

Thus, as an approximation of *E *maximizing Equation (2), we try to find $Ê=\arg\underset{E}{\max}Q\left(E\right)$, where *Q*(*E*) is the function maximizing the lower bound in Equation (4). Since higher values on *P*(*E*) are weighed more in the approximation by *Q*(*E*) for *τ *< 1, the better approximation is expected for the higher values. This property on the limited case is shown in Proposition 1. As is in the variational method and the EM algorithm, the maximum of the lower bound in Equation (4) is searched by a hill climbing from high temperature *τ *> 1.

In the hill climbing, *Q*(*X*), *Q*(Θ), and *Q*(*E*) are alternately updated from the following equations:

$$\begin{array}{c} Q\left(E\right)\propto \exp\left(\frac{1}{\tau }\int dX\int d\Theta Q\left(X\right)Q\left(\Theta \right)\log P\left(E|X,\Theta \right)\right),\\ Q\left(X\right)\propto \exp\left(\int dE\int d\Theta Q\left(E\right)Q\left(\Theta \right)\log P\left(X|\Theta ,E\right)\right),\\ Q\left(\Theta \right)\propto \exp\left(\int dE\int dXQ\left(E\right)Q\left(X\right)\log P\left(\Theta |X,E\right)\right). \end{array}$$

Gradually converging *τ *to 0, local optimum of the lower bound and corresponding *Q*(*E*), *Q*(*X*), and *Q*(Θ) are obtained.

#### Effectiveness of variational annealing

As alternatives of the variational annealing, we may consider the variational method and the EM algorithm where *X *is the set of hidden variables and *E *is handled as the set of parameters to be maximized. We show the effectiveness of variational annealing compared to the variational method and the EM algorithm under the following conditions: *P*(*X*, Θ, *E*) is factorized into *P*(*X*, *E*)*P*(Θ, *E*), and *P(E*_*i*_|*X*, Θ, *E*\{*E*_*i*_}) is given as a binomial distribution, where *E*_*i *_is the *i*th element of *E*. If factorization of *P*(*X*, Θ, *E*) = *Q*(*X*)*Q*(Θ)*Q*(*E*) is assumed in the calculation of the variational method, arg max_*E *_*Q*(*E*) is not the optimal solution of Equation (2) in general. In the EM algorithm, by allowing *E *to move around a *p*-dimensional continuous space ${\left(0,1\right)}^{p},{Ê}_{\text{EM}}=\arg\underset{E\in {\left(0,1\right)}^{m}}{\max}P\left(E\right)$ can be calculated. Let ${Ê}_{\text{EM},i}$ be the *i*th element of ${Ê}_{\text{EM}}$. Usually, ${Ê}_{\text{EM}}\text{,}i$ is mapped to 1 if ${Ê}_{\text{EM,}\;\text{i}}>0.5$ and 0 otherwise for discretizing ${Ê}_{\text{EM}}$ to the *p*-dimensional binary space {0,1}^*p*^, but such a mapping is not guaranteed to provide $\arg\underset{E\in {\left\{0,1\right\}}^{m}}{\max}P\left(E\right)$. Although other mappings can be considered, to the best of our knowledge, no mapping is guaranteed to provide the optimal solution in polynomial time of *p*.

In the following, we prove a proposition in order to show that the variational annealing possibly give the optimal solution of Equation (2) even if the factorization of $Q\left(E\right)=\prod_{i=1}^{p}Q\left(E_{i}\right)$ is additionally considered.

**Proposition 1**. *P*(*X*,Θ,*E*) *is factorized into P*(Θ,*E*)*P*(*X*,*E*), *and P*(*E*_*i*_|*X*,Θ,*E*\{*E*_*i*_}) *is given as a binomial distribution. Let *$\hat{Q}\left(E_{i}\right)$*be Q*(*E*_*i*_) *maximizing the lower bound of the variational annealing for τ *→ +0 *given by*

(5) $$\int dE_{1}...\int dE_{p}\int dX\int d\Theta Q\left(X\right)Q\left(\Theta \right)\underset{i}{\prod }Q\left(E_{i}\right)\log\frac{P\left(X,\Theta ,E\right)}{Q\left(X\right)Q\left(\Theta \right)\prod_{i}Q{\left(E_{i}\right)}^{\tau }}.$$

*Then, the set of E*_*i *_∈ {0,1} *maximizing *${Ê}_{\text{EM}}$*is *$\arg\underset{E\in {\left\{0,1\right\}}^{p}}{\max}P\left(E\right)$.

For the proof of the proposition, see Section 1 in Additional file 1. From Proposition 1, if the factorization *P*(*X*, Θ, *E*) = *P*(Θ, *E*)*P*(*X*, *E*) is satisfied and optimal *Q *functions are found, the variational annealing is guaranteed to provide the optimal solution of Equation (2) while the variational method and the EM algorithm are not. Although the factorization is not a generally satisfied property, the factorization is often assumed in approaches based on the variational method, and the assumption usually works as good approximations. Thus, the variational annealing is expected to provide the better performance than the variational method and the EM algorithm even if the factorization is not satisfied exactly.

### Procedures of variational annealing on proposed model

In the variational annealing on the proposed model, we calculate *Q *functions for hidden variables *X*, parameters Θ, and binary variables *E *iteratively while cooling temperature *τ *to zero gradually at each iteration cycle. In the following, we show the calculation procedures of *Q*(*X*), *Q*(Θ), and *Q*(*E*) on the proposed model as variational E-step, variational M-step, and variational A-step, respectively. More details of the procedures are given in Additional file 1. For the notational brevity, we denote the expectation of a value *x *with a probability distribution *Q*(*y*) as 〈*x*〉_*Q*(*y*)_.

#### Variational E-step

Parameters of *Q*(*X*) are mean of ***x***_*t*_, variance of ***x***_*t*_, and cross time variance of ***x***_*t*-1 _and ***x***_*t*_. These parameters can be calculated via variational Kalman filter by using following terms expected with *Q*(Θ)*Q*(*E*): 〈*E*^(*c*)^〉_*Q*(Θ)*Q*(*E*)_, 〈*A*〉_*Q*(Θ)*Q*(*E*)_, 〈*H*^-1 ^*A *○ *E*^(*c*)^〉_*Q*(Θ)*Q*(*E*)_, and 〈(*A *○ *E*^(*c*)^)'*H*^-1^*A *○ *E*^(*c*)^〉_*Q*(Θ)*Q*(*E*)_. For the details of variational Kalman filter, see [4]. From the parameters of *Q*(*X*), expectations of the following terms with *Q*(*X*) required in other steps are calculated: ${\langle x_{t}^{\left(c\right)}\rangle }_{Q\left(X\right)},{\langle x_{t}^{\left(c\right)}{\left(x_{t}^{\left(c\right)}\right)}^{\prime }\rangle }_{Q\left(X\right)}$, and ${\langle x_{t+1}^{\left(c\right)}{\left(x_{t}^{\left(c\right)}\right)}^{\prime }\rangle }_{Q\left(X\right)}$.

#### Variational M-step

*Q*(Θ) is factorized into ∏_*i*_*Q*(*A*_*i*_|*h*_*i*_) *Q*(*h*_*i*_)*Q*(*r*_*i*_) ∏_*j*_*Q*(*z*_*ij*_), where *A*_*i *_is a vector given by *(A*_*i*__1_, ..., *A*_*ip*_)'. From the design of the proposed model, *Q*(*A*_*i*_|*h*_*i*_), *Q*(*h*_*i*_), *Q*(*r*_*i*_), and *Q*(*z*_*ij*_) are given in the following form:

$$\begin{array}{llll} Q\left(A_{i}|h_{i}\right) & =N\left(A_{i};\mu_{A_{i}},h_{i}T_{A_{i}}^{-1}\right),\; & & \;\\ Q\left(h_{i}\right) & =IG\left(h_{i};u_{i},k_{i}\right),\; & & \;\\ Q\left(r_{i}\right) & =IG\left(r_{i};v_{i},l_{i}\right),\; & & \;\\ Q\left(z_{ij}\right) & =B\left({\zeta_{ij}}_{,0};\zeta_{ij,1}\right).\; & & \;\\ & \; & \end{array}$$

Parameters for the above functions are calculated by using ${\langle x_{t}^{\left(c\right)}\rangle }_{Q\left(X\right)},{\langle x_{t}^{\left(c\right)}{\left(x_{t}^{\left(c\right)}\right)}^{\prime }\rangle }_{Q\left(X\right)},{\langle x_{t+1}^{\left(c\right)}{\left(x_{t}^{\left(c\right)}\right)}^{\prime }\rangle }_{Q\left(X\right)},$, and ${\langle E_{ij}^{\left(c\right)}\rangle }_{Q\left(E\right)}$.

#### Variational A-step

For the calculation of *Q*(*E*), we assume the factorization of *Q*(*E*) to $\prod_{c}\prod_{ij}Q\left(E_{ij}^{\left(c\right)}\right)$ in order to make the computation tractable. $Q\left(E_{ij}^{\left(c\right)}\right)$ follows a binomial distribution that takes one with probability $e_{ij}^{\left(c\right)}$ and zero with probability $1-e_{ij}^{\left(c\right)}$, and thus the expectation of $E_{ij}^{\left(c\right)}$ with *Q*(*E*) is given by $e_{ij}^{\left(c\right)}$. For the preparation, we calculate ${\langle A\rangle }_{Q\left(\Theta \right)},{\langle H^{-1}A\rangle }_{Q\left(\Theta \right)},{\langle A^{\prime }H^{-1}A\rangle }_{Q\left(\Theta \right)},{\langle x_{t}^{\left(c\right)}\rangle }_{Q\left(X\right)},{\langle x_{t}^{\left(c\right)}{\left(x_{t}^{\left(c\right)}\right)}^{\prime }\rangle }_{Q\left(X\right)}$, and ${\langle x_{t+1}^{\left(c\right)}{\left(x_{t}^{\left(c\right)}\right)}^{\prime }\rangle }_{Q\left(X\right)}$. $Q\left(E_{ij}^{\left(c\right)}\right)$ is then iteratively calculated by using these expected terms as well as ${\langle E_{ik}^{\left(c\right)}\rangle }_{Q\left(E\right)}$ for *k *≠ *j*. A few iterations are enough for the convergence.

#### Update and selection of hyperparameters

The proposed model contains *u*_0_, *k*_0_, *v*_0_, *l*_0_, *ζ*_*i*0_, *ζ*_*i *1_, *α*_0_, and *α*_1 _as hyperparameters. *α*_0 _and *α*_1 _should be *α*_0 _<*α*_1 _as in the model setting, but this condition can be violated in the update step of the variational method. Thus, we select *α*_0 _and *α*_1 _by cross validation, and update other hyperparameters as in the variational method.

We first consider update of hyperparameters *u*_0_, *k*_0_, *v*_0_, *l*_0_, *ζ*_*i*0_, and *ζ*_*i*1 _to increase the lower bound of marginal probability. *u*_0 _and *k*_0 _are updated by maximizing the following equation:

(û0,k^0)= argmax(u0,k0) ∑i∫ Q(hi)logIG(hi;u0,k0)dhi= argmax(u0,k0)(u0-1)∑i〈loghi〉Q(hi)p+u0 logk0-k0∑i〈1∕hi〉Q(hi)p- logΓ(u0).

${û}_{0}$ and ${\hat{k}}_{0}$ are obtained by numerical optimization methods such as the Newton-Raphson method. *v*_0 _and *l*_0 _are also updated in a similar manner to *u*_0 _and *k*_0_. *ζ*_*i*0 _and *ζ*_*i*1 _are updated by solving the following equation:

$$\begin{array}{llll} \left({\hat{\zeta }}_{i0},{\hat{\zeta }}_{i1}\right) & =\arg\underset{\zeta_{i0},\zeta_{i1}}{\max}\underset{j}{\sum }\int Q\left(z_{ij}\right)\log B\left(z_{ij};\zeta_{i0},\zeta_{i1}\right)dz_{ij}\; & & \;\\ & =\arg\underset{\zeta_{i0},\zeta_{i1}}{\max}\left(\zeta_{i1}-1\right)\frac{\sum_{j}{\langle \log\left(1-z_{ij}\right)\rangle }_{Q\left(z_{ij}\right)}}{p}+\left(\zeta_{i0}-1\right)\frac{\sum_{j}{\langle \log z_{ij}\rangle }_{Q\left(z_{ij}\right)}}{p}-\log B\left(\zeta_{i0},\zeta_{i1}\right).\; & & \;\\ & \; & \end{array}$$

${\hat{\zeta }}_{i0}$ and ${\hat{\zeta }}_{i1}$ can also be obtained by the Newton-Raphson method.

For the selection of *α*_0 _and *α*_1_, we set *α*_1 _to some large value and select *α*_0 _by a leave one out cross validation procedure. For condition *c *∈ {1, 2} and time point $t\in T_{obs}^{\left(c\right)}$, we remove $y_{t}^{\left(c\right)}$ from data set ***y ***and use the data set to train the model. We calculate square sum of residues $r_{t,c}^{2}$ between $y_{t}^{\left(c\right)}$ and the prediction of $x_{t}^{\left(c\right)}$ estimated from the variational Kalman filter on the trained model given by $r_{t,c}^{2}={\left(y_{t}^{\left(c\right)}-x_{t}^{\left(c\right)}\right)}^{\prime }\left(y_{t}^{\left(c\right)}-x_{t}^{\left(c\right)}\right)$. By grid search on parameter space of *α*_0_, we select *α*_0 _that minimizes $\sum_{c=1}^{2}\sum_{t\in T_{obs}^{\left(c\right)}}r_{t,c}^{2}$.

#### Summary of procedures

The procedures for estimating parameters in the proposed model are summarized as follows:

1. Set *τ *to some large value. Also set *α*_0 _to a small value and *α*_1 _to a large value satisfying that *α*_0 _<*α*_1_.

2. Initialize other hyperparameters and hidden variables.

3. Perform the following procedures:

(a) Calculate variational M-step.

(b) Update hyperparameters.

(c) Calculate variational E-step.

(d) Calculate variational A-step.

(e) Go back to step (a) until some convergence criterion is satisfied.

4. Divide *τ *by some value > 1 such as 1.05.

5. Go back to step 3 if *τ *is larger than some very small value > 0.

In our setting, *α*_1 _is set to 1,000. For the initialization of *τ *and other hyperparameters, we use the following settings: $\tau =2.5,E_{ij}^{\left(c\right)}=0.5,u_{i}=1,k_{i}=1,v_{i}=1,l_{i}=1,\zeta_{i0}=10,\text{ and }\zeta_{i1}=10$. If $y_{t}^{\left(c\right)}$ is observed, we initialize $x_{t}^{\left(c\right)}$ with $y_{t}^{\left(c\right)}$. Otherwise, we use the linearly interpolated one.

## Results and discussion

### Performance evaluation by Monte Carlo experiments

For the evaluation of the proposed approach, we generate two linear regulatory network models with similar topological structures *G*_1 _and *G*_2 _based on a linear regulatory network model *G*_0_. *G*_0 _is prepared in the following manner: (i) a scale free network of 100 nodes and 150 edges is generated; (ii) edge directions are assigned randomly; (iii) autoloop edges are added to root nodes of the directed network; and (iv) AR coefficients for the directed edges are chosen randomly from {-0.9, -0.8, -0.7, -0.6, -0.5, 0.5, 0.6, 0.7, 0.8, 0.9}. We then generate *G*_1 _and *G*_2 _from *G*_0 _as follows: (i) autoloop edges and 70% of non-autoloop edges in *G*_0 _are used for commonly existing edges in *G*_1 _and *G*_2_; and (ii) the other 30% of non-autoloop edges are randomly assigned as either *G*_1 _or *G*_2 _specific edges. Note that AR coefficients on edges of *G*_1 _and *G*_2 _are preserved, i.e., if a regulation from gene *j *to gene *i *exists in *G*_1 _or *G*_2_, then its coefficient is the same as that of the regulation from gene *j *to gene *i *in *G*_0_.

**Figure 2:**
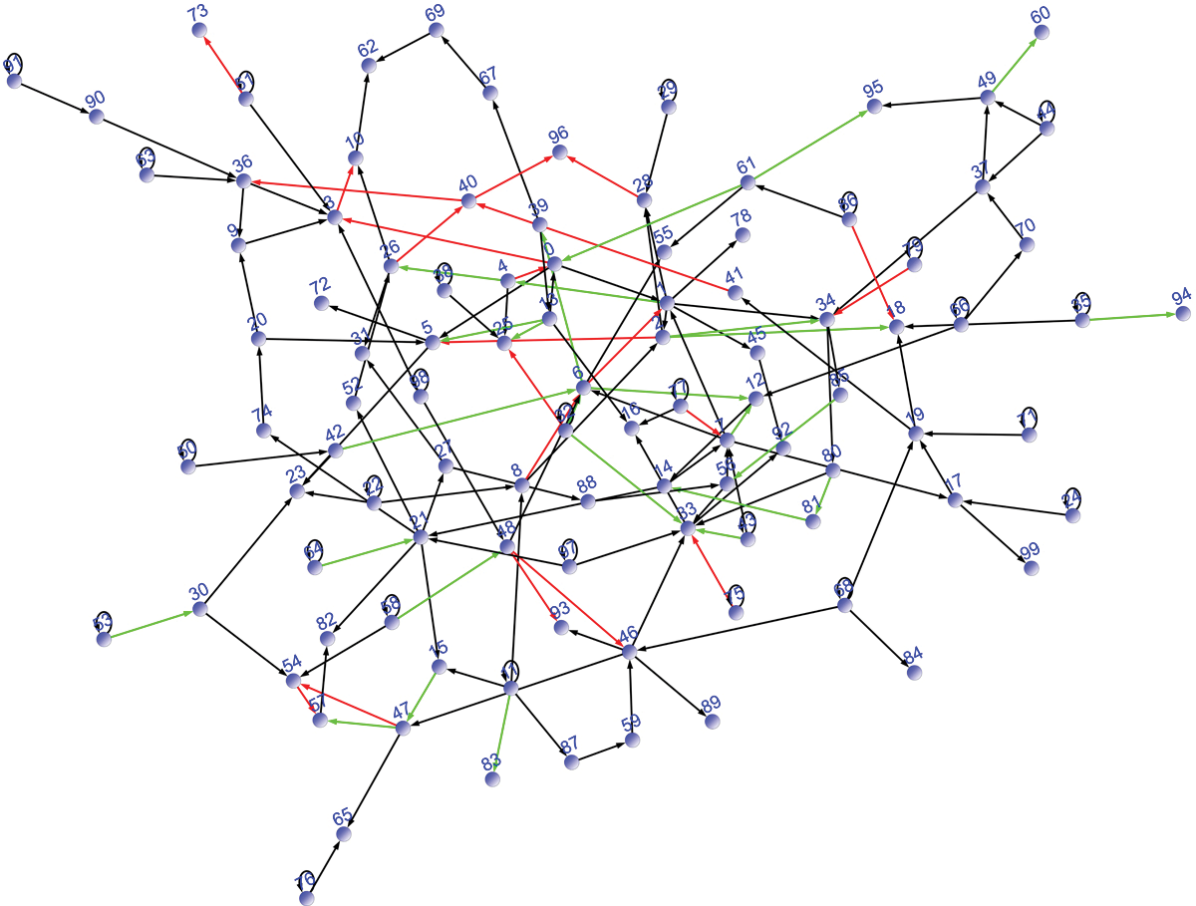
**The graph structures of *G*_1 _and *G*_2_. **Commonly regulations, *G*_1 _specific regulations, and *G*_2 _specific regulations are represented with black, red, and green arrows, respectively.

Figure 2 gives graph structure of *G*_1 _and *G*_2_, where commonly regulations, *G*_1 _specific regulations, and *G*_2 _specific regulations are represented with black, red, and green arrows, respectively.

From each of *G*_1 _and *G*_2_, we obtain two equally spaced time series data of 25 time points and 50 time points. For system noise and observation noise, normally distributed values with mean 0 and standard deviation 1 and mean 0 and standard deviation 0.1 and 1 are used, respectively. The signal-noise ratio for system noise with standard deviation 1 and observation noise with standard deviation 0.1 is 0.03dB, and the signal is bit stronger than the noise. On the other hand, for observation noise with standard deviation 1, the signal-noise ratio is -0.26dB. In the condition, the noise is stronger than the signal, and the noise level is quite high.

#### Comparison between variational annealing and EM algorithm

We first compare the performances of the proposed approach and the approach that is based on the proposed approach but uses the EM algorithm instead of the variation annealing using the equally spaced time series data of 50 and 25 time points on the system noise with standard deviation 1 and observation noise with standard deviation 0.1. From the comparison, we verify the effectiveness of the variational annealing, compared to the EM algorithm. Table 1 summarizes the results of the proposed approach and the EM algorithm based approach. For the EM algorithm, the regulation from *j *to *i *on condition *c *is considered to exist if the estimated $E_{ij}^{\left(c\right)}$ is more than 0.5.

**Table 1:** Comparison of the variation annealing (Proposed) and EM algorithm (EM) based on the proposed model

(a)						
**# of time points**	**50**			**25**		

	**# TP**	**# FP**	**PRE**	**# TP**	**# FP**	**PRE**
Proposed	295.9	41.7	0.88	238.4	71.6	0.77
EM	294.9	119.2	0.71	196.8	66.9	0.75

**(b)**						

**# of time points**	**50**			**25**		

	**# TP**	**# FP**	**PRE**	**# TP**	**# FP**	**PRE**
Proposed	39.8	13.2	0.75	23.4	20.8	0.53
EM	39.9	39.4	0.5	11.5	10.0	0.53

(a) The number of true positives (# TP) and false positives (# FP) of estimated regulations in two network models by the proposed approach and EM for equally spaced time series data. PRE denotes the precision of the results. Regulations in two networks are 305 in total. (b) The number of true positives (# TP) and false positives (# FP) of changes on regulations between two network models estimated by the proposed approach and EM for equally spaced time series data. The regulations changed in two networks are in total 47.

From the comparison, the results of the proposed approach contain more true positives than those of the EM algorithm based approach except for identifying changes on regulations for time points 50. For identifying changes on regulations, the EM algorithm based approach estimates bit more true positives than the proposed approach, but the difference is so small that it can be ignored. On the other hand, the results of the EM algorithm based approach contain more false positives than those of the proposed approach, and hence the precision of the results by the EM algorithm is worse than that of the proposed approach. Therefore, the effectiveness of the variational annealing is confirmed in the computational experiment as well.

#### Comparison between proposed approach and existing approaches

We employ the elastic net based VAR model approach [2] and the dynamic Bayesian network based approach termed G1DBN [8] as existing approaches for estimating networks from time series data. For the experiments, two versions of these approaches are considered: ENet1 and ENet2 from the elastic net based VAR model approach, and G1DBN1 and G1DBN2 from G1DBN. These approaches are different in the following point: ENet1 and G1DBN1 estimate *G*_1 _and *G*_2 _independently, i.e., for the estimation of *G*_1_, only time series data from *G*_1 _is used, while ENet2 and G1DBN2 assume that *G*_1 _and *G*_2 _have the same network structure and estimate a network by using two time series data. Thus, ENet2 and G1DBN2 are considered to more use data sample than ENet1 and G1DBN1 for network estimation, but changes on regulations between *G*_1 _and *G*_2 _are not considered. For selection of hyperparameters in ENet1 and ENet2, AICc is used [2], and for hyperparameters *α*_1 _and *α*_2 _of G1DBN1 and G1DBN2, a setting of *α*_1 _= 0.1 and *α*_2 _= 0.0059 considered in [8] is used.

For the comparison of these approaches, we focus on the following two points: the number of correctly estimated regulations and the number of correctly estimated changes on regulations. The former is usually considered for evaluating the performance of gene network estimation methods. The numbers of true positives and false negatives of the estimated regulations are summarized in Table 2(a). The precisions of the results given by

**Table 2:** A summary of results for system noise with standard deviation 1 and observation noise with standard deviation 0.1

(a)												
	**Equally spaced**						**Unequally spaced**					

**# of time points**	**50**			**25**			**50**			**25**		

	**# TP**	**# FP**	**PRE**	**# TP**	**# FP**	**PRE**	**# TP**	**# FP**	**PRE**	**# TP**	**# FP**	**PRE**
Proposed	295.9	41.7	0.88	238.4	71.6	0.77	262.4	42.1	0.86	110.7	37.2	0.75
ENet1	246.3	119.7	0.67	109.2	67	0.62	84.7	140.6	0.38	20.3	70.4	0.22
ENet2	277.9	130.9	0.68	212.8	130	0.62	169.7	241.5	0.41	65.5	132.5	0.33
G1DBN1	223.7	48	0.82	99.9	46.2	0.68	65.1	83.1	0.44	19.3	72.7	0.21
G1DBN2	268.8	83.4	0.76	188.1	64.5	0.74	134.8	104.4	0.56	46.7	85.7	0.35

**(b)**												

	**Equally spaced**						**Unequally spaced**					

**# of time points**	**50**			**25**			**50**			**25**		

	**# TP**	**# FP**	**PRE**	**# TP**	**# FP**	**PRE**	**# TP**	**# FP**	**PRE**	**# TP**	**# FP**	**PRE**
Proposed	39.8	13.2	0.75	23.4	20.8	0.53	31.2	16.5	0.65	5.5	15.9	0.26
ENet1	38.5	153.1	0.2	18.6	113	0.14	12.3	186.6	0.06	3.7	85	0.04
ENet2	-	-	-	-	-	-	-	-	-	-	-	-
G1DBN1	35.6	88.9	0.29	16.8	91.9	0.15	10.3	121.9	0.08	2.4	87.8	0.03
G1DBN2	-	-	-	-	-	-	-	-	-	-	-	-

(a) The number of true positives (# TP) and false positives (# FP) of estimated regulations in two network model by the proposed approach, ENet1, ENet2, G1DBN1, and G1DBN2 for equally and unequally spaced time series data. PRE denotes the precision of the results. Regulations in two networks are 305 in total. (b) The number of true positives (# TP) and false positives (# FP) of changes on regulations between two network models estimated by the proposed approach, ENet1, ENet2, G1DBN1, and G1DBN2 for equally and unequally spaced time series data. Since no changes are estimated by ENet2 and G1DBN2, their results are indicated by '-'. The regulations changed in two networks are in total 47.

$$\frac{\text{The}\;\text{number}\;\text{of}\;\text{true}\;\text{positives}}{\text{The}\;\text{number}\;\text{of}\;\text{true}\;\text{positives + The}\;\text{number}\;\text{of}\;\text{false}\;\text{positives}}$$

are also provided. The results are averaged on ten data sets. The number of regulations in the true network models of *G*_1 _and *G*_2 _are in total 305. For the latter point, we consider the estimated regulations existing only in one of two estimated networks as changed regulations, and check if they correctly exist only in the corresponding true network. The numbers of true positives and false negatives of the estimated changes on regulations and the precisions are summarized in Table 2(b). The results are also averaged on ten data sets. The number of true changes on regulations between in *G*_1 _and *G*_2 _are 47, i.e., the number of true positives on this case is at most 47.

For the estimation of the regulations in Table 2(a), the proposed approach outperforms other approaches in terms of true positives. The proposed approach contains more false positives than ENet1, G1DBN1, and G1DBN2 on the data of 25 time points. We further consider these cases in terms of the precision. The precisions of the proposed approach, ENet1, G1DBN1, and G1DBN2 are given by 0.77, 0.62, 0.68, and 0.74, respectively. From this analysis, the proposed approach shows better performance than ENet2, G1DBN1, and G1DBN2 on the whole. More true positives are estimated by ENet2 than ENet1, and the precisions of ENet2 tend to be better than those of ENet1. This type of relationship is also observed between G1DBN1 and G1DBN2. Also, the elastic net based VAR model approaches estimate more true positives than the approaches from G1DBN. However, in the precision, the approaches from G1DBN are better than the elastic net based VAR model approaches. From the results in Table 2(b), we see that the proposed approach estimates more true changes on regulations than ENet1 and G1DBN1 on both data of 25 and 50 time points. In addition, the results of the proposed approach contain less false positives than those of ENet1 and G1DBN1. No changes on regulations are detected in ENet2 and G1DBN2 as topological differences are ignored in these approaches. Since the proposed approach considers differences on network structures as well as uses two time series data efficiently, it can provide better results than other approaches on both estimating regulations and identifying changes on regulations.

One may think it is strange that false positives in ENet1 and G1DBN1 in Table 2(b) is more than those in Table 2(a), but this case can occur from the following reason. If a regulation exists in both of the true network models of *G*_1 _and *G*_2_, but is estimated only for *G*_1_, then the case is not counted as a false positive in Table 2(a) while it is counted as a false positive in Table 2(b). Thus, the number of false positives in Table 2(b) can be greater than those in Table 2(a).

**Figure 3:**
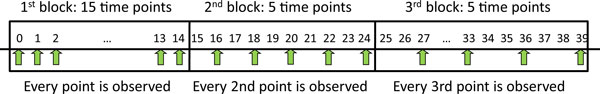
**A time point schedule on unequally spaced time series data in the Monte Carlo experiment.** Observed points in the time schedule are indicated by arrows. 15 time points are equally spaced in first block, every second point is observed in second block comprised of 5 observed time points, and every third point is observed in third block comprised of 5 observed time points.

In order to show the performance in unequally spaced time series data, we generate unequally spaced time series data of 25 and 50 observed time points. For time series data of 25 observed time points, we first generate equally spaced time series data of 40 time points and divide it into three blocks: 15 time points, 10 time points, and 15 time points. We then remove time points in the following manner: no time point is removed in the first block; one of every two time points are removed in the second block; and two of every three time points are removed in the third block. Figure 3 shows the time point schedule of time series data obtained in this process. For time series data of 50 observed time points, we first generate equally space time series data of 80 time points, divide it into three blocks: 30 time points, 20 time points, and 30 time points. Then, some time points are removed in a similar manner. We apply the proposed approach, ENet1, ENet2, G1DBN1, and G1DBN2 to the unequally spaced time series data. Results for the dataset are also summarized in Tables 2(a) and 2(b). From the comparison of results on equally and spaced time series data, the results of the proposed approach from unequally spaced time series data are worse than those from equally spaced one even with the same number of observed time points. However, results of ENet1, ENet2, G1DBN1, and G1DBN2 are worsened more than those of the proposed approach. This is probably because unequally spaced time points break their assumption, and their estimation process is misled.

We also consider the time series data with the high level noise: system noise with standard deviation 1 and observation noise with standard deviation 1. The results for the case are summarized in Tables 3(a) and 3(b). The proposed approach shows the better performance on the number of true positives and precisions than other approaches except for the identification of the changes on regulations from the equally spaced time series data of 50 time points. For equally spaced time series data of 50 time points, the number of true positives on the changes on regulations estimated by the proposed approach is more than that of G1DBN1, but less than that of ENet1. However, the precisions on the estimated changes by both ENet1 and G1DBN are much worse than the proposed approach. Thus, overall, the proposed approach is more effective than other methods. Although the proposed approach provides the better performance than other methods, the results of all the approaches are worsened due to the high level noise, and the differences on the performance among the approaches get smaller, compared to the case of observation noise with standard deviation 0.1.

**Table 3:** A summary of results for system noise with standard deviation 1 and observation noise with standard deviation 1

(a)												
	**Equally spaced**						**Unequally spaced**					

**# of time points**	**50**			**25**			**50**			**25**		

	**# TP**	**# FP**	**PRE**	**# TP**	**# FP**	**PRE**	**# TP**	**# FP**	**PRE**	**# TP**	**# FP**	**PRE**
Proposed	190.2	122.8	0.61	88.1	121.0	0.42	132.1	675.5	0.66	52.1	108.9	0.33
ENet1	110.8	136.9	0.45	30.3	75.9	0.29	32.5	133.7	0.2	7.4	75.2	0.09
ENet2	189.8	218	0.47	85.8	136.2	0.39	75.9	180.7	0.3	23.5	123.3	0.16
GIDBN1	86.6	82.6	0.51	22.6	90.8	0.2	26.3	74	0.26	7.1	71.6	0.09
GIDBN2	163.9	105.7	0.61	54.4	99	0.35	66.2	91.2	0.42	17.4	92.8	0.16

**(b)**												

	**Equally spaced**						**Unequally spaced**					

**# of time points**	**50**			**25**			**50**			**25**		

	**# TP**	**# FP**	**PRE**	**# TP**	**# FP**	**PRE**	**# TP**	**# FP**	**PRE**	**# TP**	**# FP**	**PRE**

Proposed	15.4	43.6	0.26	4.7	50.0	0.09	8.1	16.7	0.33	3.1	42.5	0.07
ENet1	16.9	184	0.08	3.8	95.4	0.04	5.2	155	0.03	1.2	81.4	0.01
ENet2	-	-	-	-	-	-	-	-	-	-	-	-
GIDBN1	14.5	125.1	0.1	3.9	105.7	0.04	4	91.9	0.04	1.7	76.8	0.02
GIDBN2	-	-	-	-	-	-	-	-	-	-	-	-

(a) The number of true positives (# TP) and false positives (# FP) of estimated regulations in two network model by the proposed approach, ENet1, ENet2, G1DBN1, and G1DBN2 for equally and unequally spaced time series data. PRE denotes the precision of the results. Regulations in two networks are 305 in total. (b) The number of true positives (# TP) and false positives (# FP) of changes on regulations between two network models estimated by the proposed approach, ENet1, ENet2, G1DBN1, and G1DBN2 for equally and unequally spaced time series data. Since no changes are estimated by ENet2 and G1DBN2, their results are indicated by '-'. The regulations changed in two networks are in total 47.

### Analysis of time series microarray data from Human small airway epithelial cells

We apply the proposed approach to two time series microarray gene expression data from normal Human small airway epithelial cells (SAECs) and SAECs treated by stimulating EGF-receptors and dosing an anticancer drug termed Gefitinib. EGF-receptors are often overexpressed in lung cancer cells such as tumoral SAECs, and a lung cancer condition is simulated in the treated SAECs by stimulating EGF-receptors. Since Gefitinib is known as a selective inhibiter of EGF-receptors, the stimulation of EGF-receptors would be counteracted by Gefitinib, and the condition of treated SACEs should be the same as that of normal SAECs in theory. However, since some gene expression patterns are different between the two conditions in practice, some unknown effects by Gefitinib may be involved in the phenomenon. Thus, we focus on changed regulations between the gene networks estimated from gene expression data in these two conditions in order to find some insights on the unknown effects of Gefitinib.

For gene set selection, we first screen 500 genes from the ranking of the gene list sorted by coefficient variation [5]. We then select 100 genes with highly varied expression profiles between normal SAECs and treated SAECs. The time series gene expression data from both types of cells are comprised of spaced 14 time points in 48 hours. The time schedule of 14 time points are {0 h, 6 h, 9 h, 12 h, 15 h, 18 h, 21 h, 24 h, 27 h, 30 h, 33 h, 36 h, 39 h, 48 h}. For the analysis on the proposed approach, we set interval on system model to three hours.

**Figure 4:**
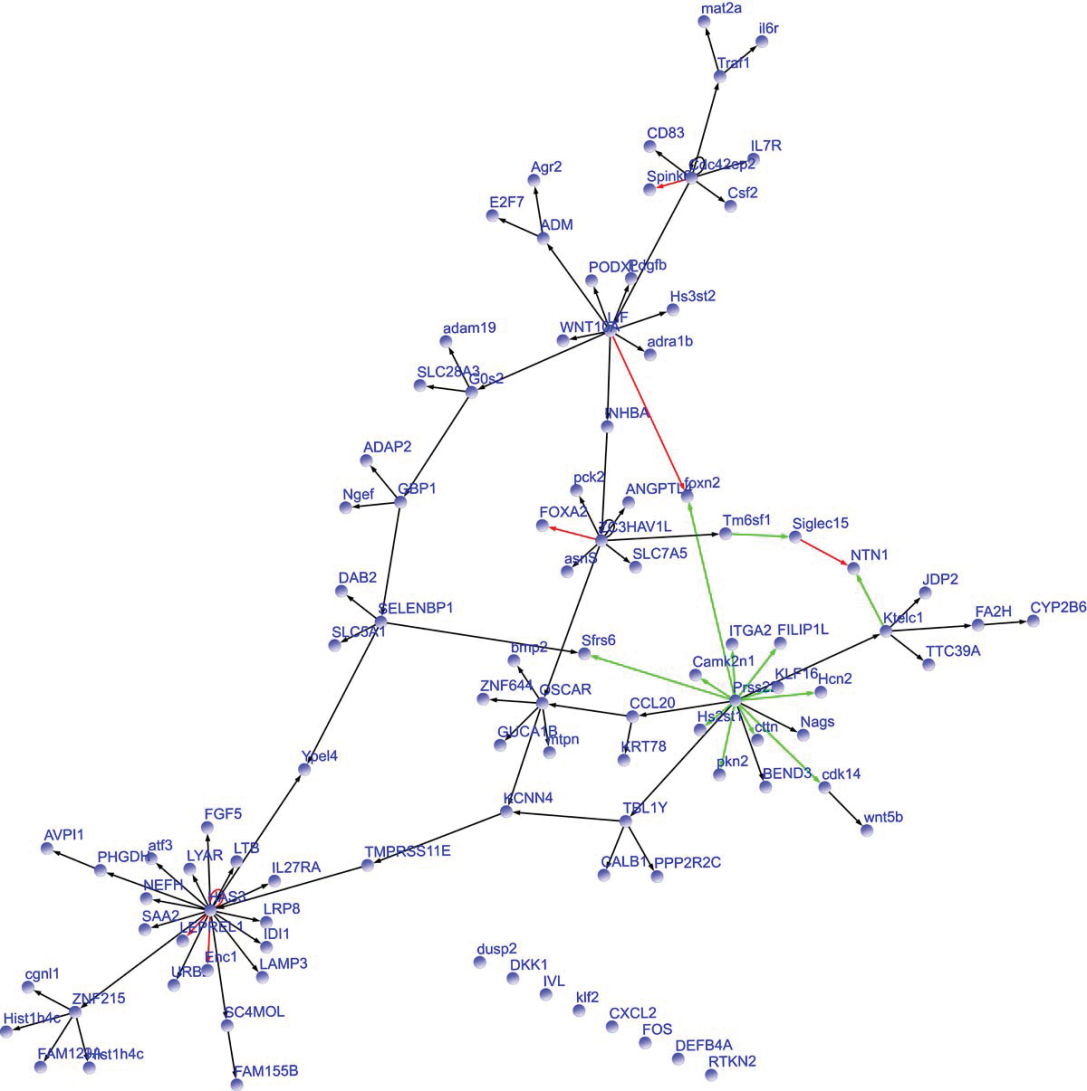
**An estimated gene network by the proposed approach from time series gene expression data on normal SAECs and treated SAECs.** In the estimated network, regulations in both conditions are in black, and regulations only in normal SAECs and treated SAECs are in red and green, respectively.

The estimated networks from time series gene expression data in normal SAECs and treated SAECs are summarized and given in Figure 4. Black arrows, red arrows, and green arrows indicate regulations in both conditions, only in normal SAECs, and only in treated SAECs, respectively. Table 4 gives a list of the estimated regulations only in normal SAECs or in treated SAEC.

**Table 4:** Changes on regulations between normal and treated SAECs

Normal SAECs	Treated SAECs
ZC3HAV1L → FOXA2	Prss22 → foxn2
LIF → foxn2	Prss22 → cdk14
Cdc42ep2 → Spink6	Prss22 → Camk2n1
Siglec15 → NTN1	Prss22 → cttn
HAS3 → HAS3	Prss22 → Sfrs6
HAS3 → Enc1	Prss22 → ITGA2
HAS3 → LEPREL1	Prss22 → pkn2
	Prss22 → Hs2st1
	Prss22 → FILIP1L
	Prss22 → Hcn2
	Prss22 → KLF16
	Ktelc1 → NTN1
	Tm6sf1 → Siglec15

Estimated regulations only in normal or treated SAECs are listed in the left side or right side, respectively.

From Table 4, we see that Prss22 is involved in most of the regulations only in treated SAECs. Prss22 is a tryptase, one of serine proteases, and its relationship with the airways is suggested by a report about its expression in the airways in a developmentally regulated manner. Tryptase is a potent mitogen of fibroblast [15], and it is reported that the increase and activation of fibroblast are promoted in lung cells under the condition of interstitial pneumonia. Interstitial pneumonia is known as a side effect of Gefitinib, and these findings suggest that Prss22 is an off-target of Gefitinib and is possibly related to the side effect of Gefitinib. Also, FILIP1L, a gene estimated as one of targets of Prss22 in treated SAECs, is reported as an inhibitor of cell proliferation of fibroblast [16]. This observation supports the relation between Prss22 and fibroblast as well.

We also focus on other several genes related to changes on regulations in normal and treated SAECs. LIF, leukemia inhibitory factor, is known to affect cell growth and development. Gefitinib is also known to be effective for acute myelogenous leukemia via Sky, which is an off-target gene of Gefitinib [17]. In addition, Wang *et al. *reported that LIF prolongs the cell cycle of stem cells on acute myelogenous leukemia lines [18]. Although, to the best of our knowledge, no direct influence from Gefitinib to LIF is reported, the above facts suggest some relation between Gefitinib and LIF, and support changes on regulations of LIF between normal and treated SAECs.

Heikema *et al. *reported that Human Siglecs, Siglecs-14, -15, and -16 interact with transmembrain adaptor proteins containing the immunoreceptor tyrosine-based activation motif such as DAP12 [19], and therefore they potentially mediate the activation of intracellular signaling. Gefitinib is a selective inhibitor of tyrosine kinase, and the inhibition of tyrosine kinase is considered to affect the regulations around Siglec-15.

Although the stimulation of EGF-receptors in the treated SAECs is considered to be counteracted by Gefitinib, the expressions of some genes may be affected by the stimulation in practical conditions. HAS3 is related to synthesis of the unbranched glycosaminoglycan hyaluronic acid and is reported to be up-regulated by EGF [20]. The stimulation of EGF-receptors affects the amount of EGF taken into cells, and hence the stimulation is expected to cause the changes on the regulations around HAS3. Foxn2 is a member of family of Fox proteins. Fox proteins are known to play important roles on controlling the expressions of genes related to cell growth, proliferation, and differentiation. Some members of Fox family are related to EFG-receptors, e.g., the expression of Foxn1 is suppressed by EGF-receptor signaling [21] although no direct relation between EGF-receptors and Foxn2 is found. FOXA2 is also a member of family of Fox proteins. EGF-receptor signaling is known to decrease the expression of FOXA, which prevents the mucus production [22]. [23] reported the relation between EGF-receptors and FOXA2 in the airways of asthmatic patients. Thus, it appears that the change on the regulation related to FOXA2 is caused by the stimulation of EGF-receptors.

## Conclusions

We proposed the new computational model that is based on VAR-SSM and estimates gene networks from time series data on normal and treated conditions as well as identifies changes regulations by the treatment. Unlike many of existing gene network estimation approaches assuming equally spaced time points, our approach can handle unequally spaced time series data. The efficient use of time series data is achieved by representing the presence of regulations on each condition with hidden binary variables. Since finding the optimal configuration of the hidden binary variables on the proposed model is computationally in tractable, we derive the extended variational annealing method in order to address the problem as the alternative method.

In the Monte Carlo experiments, we use equally and spaced time series data from synthetically generated two regulatory networks whose structures are different in several regulations, and verified the effectiveness of the proposed model in both estimation of regulations and changes on regulations between the two conditions, compared to existing methods.

As the real data application, we use the proposed approach to analyze two time series data from normal SAECs and SAECs treated by stimulating EGF-receptors and dosing Gefitinib. From genes related to changes on regulations by the treatment, we find possible off-target genes of Gefitinib, and one of these genes is suggested to be related to a factor of interstitial pneumonia, which is known as a side effect of Gefitinib. In this study, we consider changes on regulations in two conditions, but the proposed approach can be extended to identifying changes among more than two conditions.

## Competing interests

The authors declare that they have no competing interests.

## Authors' contributions

KK, SI, RY, and SM designed the approach to identify the changes on regulations by the cell treatment to SAECs. KK, SI, and AF contributed to the statistical modeling for the approach, and devised the details of methodologies for estimating the proposed model. MY and NG carried out the microarray experiment for measuring time series the gene expression data on normal and treated SAECs.

## Supplementary Material

Additional file 1 **Proof of Proposition 1 and more details on the procedures of variational annealing.** A proof of Proposition 1 and more details on the procedures of variational annealing on the proposed model are described. Click here for file
