# Supplementary material for: Identifying regulational alterations in gene regulatory networks by state space representation of vector autoregressive models and variational annealing
Source: BMC Genomics. 2012 Jan 17;13(Suppl 1):S6. doi: 10.1186/1471-2164-13-S1-S6 (PMC3587380; doi:10.1186/1471-2164-13-S1-S6)
Supplement: Additional file 1 — Proof of Proposition 1 and more details on the procedures of variational annealing. A proof of Proposition 1 and more details on the procedures of variational annealing on the proposed model are described. [file 1471-2164-13-S1-S6-S1.pdf]

Additional File for Identifying Regulational Alterations in Gene Regulatory Networks by State Space Representation of Vector Autoregressive Models and Variational Annealing

Kaname Kojima[1] kaname@ims.u-tokyo.ac.jp
Seiya Imoto[1] imoto@ims.u-tokyo.ac.jp
Rui Yamaguchi[1] ruiy@ims.u-tokyo.ac.jp
André Fujita[2] fujita@ime.usp.br
Mai Yamauchi[1] cyowako@ims.u-tokyo.ac.jp
Noriko Gotoh[1] ngotoh@ims.u-tokyo.ac.jp
Satoru Miyano[1] miyano@ims.u-tokyo.ac.jp

[1]Human Genome Center, Institute of Medical Science, University of Tokyo, 4-6-1 Shirokanedai, Minato-ku, Tokyo 108-8639, Japan
[2]Institute of Mathematics and Statistics, University of São Paulo, Rua do Matão, 1010, São Paulo 05508-090, Brazil

1 Proof of Proposition 1 in Main Manuscript

We give a proof of Proposition 1 in the main manuscript.

Proposition 1. *$P(X, \Theta, E)$ is factorized into $P(\Theta, E)P(X, E)$, and $P(E_i|X, \Theta, E \setminus \{E_i\})$ is given as a binomial distribution. Let $\hat{Q}(E_i)$ be $Q(E_i)$ maximizing the lower bound of the variational annealing for $\tau \to +0$ given by*

$$\int dE_1 \cdots \int dE_p \int dX \int d\Theta Q(X)Q(\Theta) \prod_i Q(E_i) \log \frac{P(X, \Theta, E)}{Q(X)Q(\Theta)\prod_i Q(E_i)^\tau}. \tag{1}$$

Then, the set of $E_i \in \{0, 1\}$ maximizing $\hat{Q}(E_i)$ is $\arg\max_{E\in\{0,1\}^p} P(E)$.

Proof. We consider $Q(E_i)$, $Q(\Theta)$, and $Q(X)$ maximizing Equation (1) for $\tau \to +0$. Since $Q(E_i)^\tau$ is proportional to a binomial distribution, $Q(E_i)$ converges to the Dirac delta function $\delta(E_i, \hat{E}_i)$ with some $\hat{E}_i \in \{0, 1\}$ for $\tau \to +0$. For $Q(E_i) = \delta(E_i, \hat{E}_i)$ and $\tau \to +0$, $Q(\Theta)$ and $Q(X)$ maximizing Equation (1) are respectively given as $P(\Theta|\hat{E})$ and $P(X|\hat{E})$, where $\hat{E}$ is the set of $\hat{E}_i$.

On the other hand, for $Q(\Theta) = P(\Theta|\hat{E})$, $Q(X) = P(X|\hat{E})$, and $\tau \to +0$, $Q(E_i)$ converges to $\delta(E_i, E_i^*)$ with some $E_i^* \in \{0, 1\}$, and Equation (1) is given by

$$\begin{aligned}
&\int dE_1 \cdots \int dE_p \int dX \int d\Theta \prod_i \delta(E_i, E_i^*) P(X|\hat{E})P(\Theta|\hat{E}) \log \frac{P(X|E)P(\Theta|E)P(E)}{P(X|\hat{E})P(\Theta|\hat{E})} \\
= &\int P(X|\hat{E}) \log \frac{P(X|E^*)}{P(X|\hat{E})} dX + \int P(\Theta|\hat{E}) \log \frac{P(\Theta|E^*)}{P(\Theta|\hat{E})} d\Theta + \log P(E^*),
\end{aligned} \tag{2}$$

where E^* is the set of E_i^*. E_i^* maximizing Equation (2) is $\hat{E}_i$, and for $E_i^* = \hat{E}_i$, Equation (2) amounts to $\log P(\hat{E})$. Therefore, $Q(E_i)$, $Q(\Theta)$, and $Q(X)$ maximizing Equation (1) are respectively $\delta(E_i, \hat{E}_i)$, $P(\Theta|\hat{E})$, and $P(X|\hat{E})$ with $\hat{E} = \arg\max_{E\in\{0,1\}^p} P(E)$. □

2 More Details on Procedures of Variational Annealing on Proposed Model

In the variational annealing on the proposed model, we calculate Q functions for hidden variables X, parameters Θ, and binary variables E iteratively while cooling temperature τ to zero gradually at each iteration cycle. Here, we show the calculation procedures of $Q(X)$, $Q(\Theta)$, and $Q(E)$ as variational E-step, variational M-step, and variational A-step, respectively under the complete likelihood of the proposed model:.

$$\begin{aligned} P(Y, X, \Theta, E) &= \prod_{c=1}^{2} \prod_{t \in \mathcal{T}^{(c)}} \frac{|H|^{-1/2}}{\sqrt{2\pi}^{p}} \exp\Big\{ -\frac{1}{2}(\boldsymbol{x}_t^{(c)} - A \circ E^{(c)} \boldsymbol{x}_{t-1}^{(c)})' H^{-1} (\boldsymbol{x}_t^{(c)} - A \circ E^{(c)} \boldsymbol{x}_{t-1}^{(c)}) \Big\} \\ &\times \prod_{t \in \mathcal{T}_{obs}^{(c)}} \frac{|R|^{-1/2}}{\sqrt{2\pi}^{p}} \exp\Big\{ -\frac{1}{2}(\boldsymbol{y}_t^{(c)} - \boldsymbol{x}_t^{(c)})' R^{-1} (\boldsymbol{y}_t^{(c)} - \boldsymbol{x}_t^{(c)}) \Big\} P(\Theta, E), \end{aligned}$$

For the notational brevity, we denote the expectation of a value x with a probability distribution $Q(y)$ as $\langle x \rangle_{Q(y)}$.

2.1 Variational E-step

The proposed model is considered as the state space model in terms of hidden variables $X = \{\boldsymbol{x}_t^{(c)}\}$. In the state space model, system matrix is given by $A \circ E^{(c)}$, and observation matrix is a p-dimensional identity matrix. Therefore, the parameters of $Q(X)$ are mean of $\boldsymbol{x}_t$, variance of $\boldsymbol{x}_t$, and cross time variance of $\boldsymbol{x}_{t-1}$ and $\boldsymbol{x}_t$. These parameters can be calculated via variational Kalman filter by using following terms expected with $Q(\Theta)Q(E)$: $\langle E^{(c)} \rangle_{Q(\Theta)Q(E)}$, $\langle A \rangle_{Q(\Theta)Q(E)}$, $\langle H^{-1} A \circ E^{(c)} \rangle_{Q(\Theta)Q(E)}$, and $\langle (A \circ E^{(c)})' H^{-1} A \circ E^{(c)} \rangle_{Q(\Theta)Q(E)}$. For the details of variational Kalman filter, see Chapter 5 of [1]. Let mean of $\boldsymbol{x}_t$, variance of $\boldsymbol{x}_t$, and cross time variance of $\boldsymbol{x}_{t-1}$ and $\boldsymbol{x}_t$ be $\boldsymbol{\mu}_{\boldsymbol{x}_t}$, Σ_t, and $\Sigma_{t,t-1}$, respectively. From the parameters of $Q(X)$, expectations of$\boldsymbol{x}_t^{(c)}$, $\boldsymbol{x}_t^{(c)} \left(\boldsymbol{x}_t^{(c)} \right)'$, and $\boldsymbol{x}_{t+1}^{(c)} \left(\boldsymbol{x}_t^{(c)} \right)'$ with $Q(X)$ required in other steps are calculated as follows:

$$\begin{aligned} \langle \boldsymbol{x}_t^{(c)} \rangle_{Q(X)} &= \boldsymbol{\mu}_{\boldsymbol{x}_t}, \\ \langle \boldsymbol{x}_t^{(c)} \left(\boldsymbol{x}_t^{(c)} \right)' \rangle_{Q(X)} &= \boldsymbol{\mu}_{\boldsymbol{x}_t} (\boldsymbol{\mu}_{\boldsymbol{x}_t})' + \Sigma_t, \\ \langle \boldsymbol{x}_t^{(c)} \left(\boldsymbol{x}_{t-1}^{(c)} \right)' \rangle_{Q(X)} &= \boldsymbol{\mu}_{\boldsymbol{x}_t} (\boldsymbol{\mu}_{\boldsymbol{x}_{t-1}})' + \Sigma_{t,t-1}. \end{aligned}$$

2.2 Variational M-step

$Q(\Theta)$ is factorized into $\prod_i Q(A_i|h_i)\, Q(h_i) Q(r_i) \prod_j Q(z_{ij})$, where A_i is a vector given by $(A_{i1}, \ldots, A_{ip})'$. From the design of the proposed model, $Q(A_i|h_i)$, $Q(h_i)$, $Q(r_i)$, and $Q(z_{ij})$ are given in the following form:

$$\begin{aligned} Q(A_i|h_i) &= \mathcal{N}(A_i; \boldsymbol{\mu}_{A_i}, h_i T_{A_i}^{-1}), \\ Q(h_i) &= \mathcal{IG}(h_i; u_i, k_i), \\ Q(r_i) &= \mathcal{IG}(r_i; v_i, l_i), \\ Q(z_{ij}) &= \mathcal{B}(\zeta_{ij,0}; \zeta_{ij,1}). \end{aligned}$$

Here, T_{A_i} is a matrix given by $\sum_{c=1}^{2} \sum_{t=1}^{T^{(c)}-1} \langle \boldsymbol{x}_t^{(c)} \left(\boldsymbol{x}_t^{(c)} \right)' \rangle_{Q(X)} \circ \langle E_i^{(c)} (E_i^{(c)})' \rangle_{Q(E)}$, where $E_i^{(c)}$ is a vector given by $(E_{i1}^{(c)}, \ldots, E_{ip}^{(c)})'$, and $\boldsymbol{\mu}_{A_i}$ is given by $T_{A_i}^{-1} \sum_{c=1}^{2} \sum_{t=1}^{T^{(c)}-1} \langle \boldsymbol{x}_{t+1}^{(c)} \left(\boldsymbol{x}_t^{(c)} \right)' \rangle_{Q(X)} \langle E_i^{(c)} \rangle_{Q(E)}$.

u_i, k_i, v_i, and l_i are given as follows:

$$
\begin{aligned}
u_i &= u_0 + \frac{1}{2}\sum_{c=1}^{2}(T^{(c)} - 1), \\
k_i &= k_0 + \frac{1}{2}(\sum_{c=1}^{2}\sum_{t=2}^{T^{(c)}}\langle\left(\boldsymbol{x}_{t,i}^{(c)}\right)^2\rangle_{Q(X)} - \boldsymbol{\mu}_{A_i}' T_{A_i}\boldsymbol{\mu}_{A_i}), \\
v_i &= v_0 + \frac{1}{2}\sum_{c=1}^{2}|\mathcal{T}_{obs}^{(c)}|, \\
l_i &= l_0 + \frac{1}{2}(\sum_{c=1}^{2}\sum_{t\in\mathcal{T}_{obs}^{(c)}}\left(\left(\boldsymbol{y}_{t,i}^{(c)}\right)^2 - 2\boldsymbol{y}_{t,i}^{(c)}\langle\boldsymbol{x}_{t,i}^{(c)}\rangle_{Q(X)} + \langle\left(\boldsymbol{x}_{t,i}^{(c)}\right)^2\rangle_{Q(X)}\right),
\end{aligned}
$$

where $\boldsymbol{x}_{t,i}^{(c)}$ and $\boldsymbol{y}_{t,i}^{(c)}$ are the ith element of $\boldsymbol{x}_t^{(c)}$ and $\boldsymbol{y}_t^{(c)}$, respectively.

$$
\begin{aligned}
\zeta_{ij,0} &= \zeta_{i0} + \langle E_{ij}^{(1)}\rangle_{Q(E)}\langle E_{ij}^{(2)}\rangle_{Q(E)} + (1 - \langle E_{ij}^{(1)}\rangle_{Q(E)})(1 - \langle E_{ij}^{(2)}\rangle_{Q(E)}) \\
\zeta_{ij,1} &= \zeta_{i1} + (1 - \langle E_{ij}^{(1)}\rangle_{Q(E)})\langle E_{ij}^{(2)}\rangle_{Q(E)} + \langle E_{ij}^{(1)}\rangle_{Q(E)}(1 - \langle E_{ij}^{(2)}\rangle_{Q(E)}).
\end{aligned}
$$

By using the parameters, we consider the following expectations required for the calculation of variational E-step: $\langle E^{(c)}\rangle_{Q(\Theta)Q(E)}$, $\langle A\rangle_{Q(\Theta)Q(E)}$, $\langle H^{-1}A \circ E^{(c)}\rangle_{Q(\Theta)Q(E)}$, and $\langle (A \circ E^{(c)})' H^{-1} A \circ E^{(c)}\rangle_{Q(\Theta)Q(E)}$. $\langle E^{(c)}\rangle_{Q(E)}$ is given in the procedure of variational A-step, $\langle A\rangle_{Q(\Theta)}$ is given by $(\boldsymbol{\mu}_{A_1}, \ldots, \boldsymbol{\mu}_{A_p})'$, and $\langle H^{-1}A \circ E^{(c)}\rangle_{Q(\Theta)Q(E^{(c)})}$ is given by

$$
\langle H^{-1}A \circ E^{(c)}\rangle_{Q(\Theta)Q(E^{(c)})} = \langle H^{-1}\rangle_{Q(\Theta)}\langle A\rangle_{Q(\Theta)} \circ \langle E^{(c)}\rangle_{Q(E)},
$$

where $\langle H^{-1}\rangle_{Q(\Theta)}$ is given by diag$[\langle 1/h_i\rangle_{Q(h_i)}]$ and $\langle 1/h_i\rangle_{Q(h_i)} = u_i/k_i$. Finally, $\langle (A \circ E^{(c)})' H^{-1} A \circ E^{(c)}\rangle_{Q(\Theta)Q(E)}$ is given by

$$
\langle (A \circ E^{(c)})' H^{-1} A \circ E^{(c)}\rangle_{Q(\Theta)Q(E)} = \sum_i \left(\langle 1/h_i\rangle_{Q(h_i)}\boldsymbol{\mu}_{A_i}\boldsymbol{\mu}_{A_i}' + T_{A_i}\right) \circ \langle E_i^{(c)}(E_i^{(c)})'\rangle_{Q(E)}.
$$

2.3 Variational A-step

For the calculation of $Q(E)$, we assume the factorization of $Q(E)$ to $\prod_c \prod_{ij} Q(E_{ij}^{(c)})$ in order to make the computation tractable. The likelihood with respect to $E_{ij}^{(c)}$ forms a binomial distribution. Note that $(E_{ij}^{(c)})^2$ is considered as $E_{ij}^{(c)}$ because $(E_{ij}^{(c)})^2 = E_{ij}^{(c)}$ holds for $E_{ij}^{(c)} = 0$ or 1. Thus, $Q(E_{ij}^{(c)})$ is given by a binomial distribution that takes one with probability $e_{ij}^{(c)}$ and 0 with probability $1 - e_{ij}^{(c)}$, and hence the expectation of $E_{ij}^{(c)}$ on $Q(E)$ is give as $e_{ij}^{(c)}$. For the preparation, we calculate $\langle A\rangle_{Q(\Theta)}$, $\langle H^{-1}A\rangle_{Q(\Theta)}$, $\langle A'H^{-1}A\rangle_{Q(\Theta)}$, $\langle \boldsymbol{x}_t^{(c)}\rangle_{Q(X)}$, $\langle \boldsymbol{x}_t^{(c)}\left(\boldsymbol{x}_t^{(c)}\right)'\rangle_{Q(X)}$, and $\langle \boldsymbol{x}_{t+1}^{(c)}\left(\boldsymbol{x}_t^{(c)}\right)'\rangle_{Q(X)}$. $Q(E_{ij}^{(c)})$ is then iteratively calculated by using these expectations as well as the expectations $E_{ik}^{(c)}$ for $k \neq j$ on $Q(E)$. A few iterations are enough for the convergence. Let $w_{ij}^{(c)}$ be the ith element of $\sum_{t=1}^{T^{(c)}-1}\langle \boldsymbol{x}_{t+1}^{(c)}\left(\boldsymbol{x}_t^{(c)}\right)'\rangle_{Q(X)}\boldsymbol{\mu}_{A_i}$ and $M_{ijk}^{(c)}$ be the (j,k)th element of

$$
\sum_{t=1}^{T^{(c)}-1}\langle \boldsymbol{x}_t^{(c)}\left(\boldsymbol{x}_t^{(c)}\right)'\rangle_{Q(X)}\boldsymbol{\mu}_{A_i}\boldsymbol{\mu}_{A_i} + T_{A_i}.
$$

Without loss of generality, we consider the calculation of $e_{ij}^{(c)}$ for $c = 1$. $e_{ij}^{(1)}$ is given by $\frac{\exp(\frac{1}{\tau}d_{ij}^{(1)})}{\left(1+\exp(\frac{1}{\tau}d_{ij}^{(1)})\right)}$, where

$$d_{ij}^{(1)} = w_{ij}^{(1)} + \frac{1}{2}M_{ijj}^{(1)} + \sum_{j\neq k} E_{ik}^{(1)} M_{ijk}^{(1)} + \log \sum_{E_{ij}^{(2)}=0}^{1} \phi(E_{ij}^{(1)}, E_{ij}^{(2)})\mathcal{N}(0,\alpha_1)^{F_{ij}}\mathcal{N}(0,\alpha_0)^{1-F_{ij}}.$$

By using the obtained $Q(E)$, we consider the expectations $\langle E_{ij}^{(c)}\rangle_{Q(E)}$ and$\langle E_i^{(c)}(E_i^{(c)})'\rangle_{Q(E)}$ required in the calculations of variational-E step and M-step. $\langle E_{ij}^{(c)}\rangle_{Q(E)}$ is given by $e_{ij}^{(c)}$, and the (j,k)th element of $\langle E_i^{(c)}(E_i^{(c)})'\rangle_{Q(E)}$ is given by $e_{ij}^{(c)}$ if $j = k$ holds and $e_{ij}^{(c)}e_{ik}^{(c)}$ otherwise.

3 More Details on Update of Hyperparameters

We show more details of updating hyperparamers u_0, k_0, v_0, l_0, ζ_{i0}, and ζ_{i1} by using the Newton-Raphson method. u_0 and k_0 are updated by maximizing the following equation:

$$\begin{aligned}(\hat{u}_0, \hat{k}_0) &= \arg\max_{(u_0,k_0)} \sum_i \int Q(h_i)\log \mathcal{IG}(h_i; u_0, k_0)dh_i \\ &= \arg\max_{(u_0,k_0)} (u_0 - 1)\frac{\sum_i \langle \log h_i\rangle_{Q(h_i)}}{p} + u_0 \log k_0 - k_0 \frac{\sum_i \langle 1/h_i\rangle_{Q(h_i)}}{p} - \log\Gamma(u_0),\end{aligned}$$

where $\langle \log h_i\rangle_{Q(h_i)}$ is given by $\log(k_i) - \psi(u_i)$ with the digamma function $\psi(\cdot)$. $\hat{u}_0$ and $\hat{k}_0$ are obtained by the Newton-Raphson method. Let $\boldsymbol{\gamma}$ be a vector $(u_0, k_0)'$ and $f(\boldsymbol{\gamma})$ a function given by

$$f(\boldsymbol{\gamma}) = (u_0 - 1)\frac{\sum_i \langle \log h_i\rangle_{Q(h_i)}}{p} + u_0 \log k_0 - k_0 \frac{\sum_i \langle 1/h_i\rangle_{Q(h_i)}}{p} - \log\Gamma(u_0).$$

In the Newton-Raphson method, $\hat{u}_0$ and $\hat{k}_0$ are obtained by iteratively updating the following function:

$$\boldsymbol{\gamma}^{s+1} = \boldsymbol{\gamma}^s - \left(\left.\frac{\partial^2 f(\boldsymbol{\gamma})}{\partial\boldsymbol{\gamma}\partial\boldsymbol{\gamma}'}\right|_{\boldsymbol{\gamma}=\boldsymbol{\gamma}^s}\right)^{-1} \left.\frac{\partial f(\boldsymbol{\gamma})}{\partial\boldsymbol{\gamma}}\right|_{\boldsymbol{\gamma}=\boldsymbol{\gamma}^s}.$$

Gradient and Hessian matrix of $f(\boldsymbol{\gamma})$ are given by

$$\frac{\partial f(\boldsymbol{\gamma})}{\partial\boldsymbol{\gamma}} = \begin{pmatrix} -\psi(u_0) + \log k_0 + \frac{\sum_j \langle \log(1-z_{ij})\rangle_{Q(z_{ij})}}{p} \\ u_0/k_0 - \frac{\sum_i \langle 1/h_i\rangle_{Q(h_i)}}{p} \end{pmatrix},$$

and

$$\frac{\partial^2 f(\boldsymbol{\gamma})}{\partial\boldsymbol{\gamma}\partial\boldsymbol{\gamma}'} = \begin{pmatrix} -\psi_1(u_0) & 1/k_0 \\ 1/k_0 & -u_0/k_0^2 \end{pmatrix},$$

where $\psi_1(\cdot)$ is the trigamma function. v_0 and l_0 are also updated in a similar manner to u_0 and k_0. ζ_{i0} and ζ_{i1} are updated by solving the following equation:

$$\begin{aligned}(\hat{\zeta}_{i0}, \hat{\zeta}_{i1}) &= \arg\max_{\zeta_{i0},\zeta_{i1}} \sum_j \int Q(z_{ij})\log \mathcal{B}(z_{ij}; \zeta_{i0}, \zeta_{i1})dz_{ij} \\ &= \arg\max_{\zeta_{i0},\zeta_{i1}} (\zeta_{i1} - 1)\frac{\sum_j \langle \log(1 - z_{ij})\rangle_{Q(z_{ij})}}{p} + (\zeta_{i0} - 1)\frac{\sum_j \langle \log z_{ij}\rangle_{Q(z_{ij})}}{p} - \log B(\zeta_{i0}, \zeta_{i1}).\end{aligned}$$

Let $\boldsymbol{\zeta}$ be a vector $(\zeta_{i0}, \zeta_{i1})'$ and $g(\boldsymbol{\zeta})$ a function given by

$$g(\boldsymbol{\zeta}) = (\zeta_{i1} - 1)\frac{\sum_j \langle \log(1 - z_{ij}) \rangle_{Q(z_{ij})}}{p} + (\zeta_{i0} - 1)\frac{\sum_j \langle \log z_{ij} \rangle_{Q(z_{ij})}}{p} - \log B(\zeta_{i0}, \zeta_{i1}).$$

In the Newton-Raphson method, $\hat{\zeta}_{i0}$ and $\hat{\zeta}_{i1}$ are obtained by iteratively updating the following function:

$$\boldsymbol{\zeta}^{s+1} = \boldsymbol{\zeta}^{s} - \left(\left. \frac{\partial^2 g(\boldsymbol{\zeta})}{\partial \boldsymbol{\zeta} \partial \boldsymbol{\zeta}'} \right|_{\boldsymbol{\zeta} = \boldsymbol{\zeta}^s} \right)^{-1} \left. \frac{\partial g(\boldsymbol{\zeta})}{\partial \boldsymbol{\zeta}} \right|_{\boldsymbol{\zeta} = \boldsymbol{\zeta}^s},$$

where gradient and Hessian matrix of $g(\boldsymbol{\zeta})$ are given by

$$\frac{\partial g(\boldsymbol{\zeta})}{\partial \boldsymbol{\zeta}} = \begin{pmatrix} \psi(\zeta_{i0} + \zeta_{i1}) - \psi(\zeta_{i0}) + \frac{\sum_j \langle \log z_{ij} \rangle_{Q(z_{ij})}}{p} \\ \psi(\zeta_{i0} + \zeta_{i1}) - \psi(\zeta_{i1}) + \frac{\sum_j \langle \log(1 - z_{ij}) \rangle_{Q(z_{ij})}}{p} \end{pmatrix},$$

and

$$\frac{\partial^2 g(\boldsymbol{\zeta})}{\partial \boldsymbol{\zeta} \partial \boldsymbol{\zeta}'} = \begin{pmatrix} \psi_1(\zeta_{i0} + \zeta_{i1}) - \psi_1(\zeta_{i0}) & \psi_1(\zeta_{i0} + \zeta_{i1}) \\ \psi_1(\zeta_{i0} + \zeta_{i1}) & \psi_1(\zeta_{i0} + \zeta_{i1}) - \psi_1(\zeta_{i1}) \end{pmatrix}.$$

References

[1] Beal, M.J. (2003) *Variational Algorithms for Approximate Bayesian Inference*, PhD Thesis, Gatsby Computational Neuroscience Unit, University College London, London, UK.
